# The need for evolutionary theory in cancer research

**DOI:** 10.1007/s10654-022-00936-8

**Published:** 2022-11-17

**Authors:** Amy M. Boddy

**Affiliations:** 1 Department of Anthropology, University of California Santa Barbara, Santa Barbara, CA USA; 2 Arizona Cancer and Evolution Center, Arizona State University, Tempe, AZ USA

**Keywords:** Cancer, Somatic mutations, Evolutionary theory, Peto’s paradox

## Abstract

Sir Richard Peto is well known for proposing puzzling paradoxes in cancer biology—some more well-known than others. In a 1984 piece, Peto proposed that after decades of molecular biology in cancer research, we are still ignorant of the biology underpinning cancer. Cancer is a product of somatic mutations. How do these mutations arise and what are the mechanisms? As an epidemiologist, Peto asked if we really need to understand mechanisms in order to prevent cancer? Four decades after Peto’s proposed ignorance in cancer research, we can simply ask, are we still ignorant? Did the great pursuit to uncover mechanisms of cancer eclipse our understanding of causes and preventions? Or can we get closer to treating and preventing cancer by understanding the underlying mechanisms that make us most vulnerable to this disease?

There is a danger that too great a commitment to the search for mechanisms will divert attention from the search for causes, and an Encyclopaedia of Ignorance, might be a good place to complain about this.

## Cancer research needs theory

It is well-timed that Sir Richard Peto’s witty, and insightful article on the “The need for ignorance in cancer research” has the opportunity to be republished [1]. In this piece, Peto boldly takes a reductionist approach to cancer research. Here he proposes a ‘black box strategy’ for epidemiology—in which he suggests the correlations of the cancer risk can provide more practical advances in disease prevention. This black box epidemiology approach is presented in opposition to studying the molecular mechanisms responsible for cancer.

This indicates that there are two alternative or rather complementary approaches to the prevention of cancer, the mechanistic strategy and the black box strategy

It’s spectacular to me that even in 1984, with copious unknowns in cancer biology, Peto (and others) had a considerable number of valuable predictions that have been upheld over the years. Much of Peto’s work has remained relevant for almost 40 years. Without proposed mechanisms of action or even comparative cancer data, Peto proposed his famous paradox on body size in animals and cancer risk [2]. Myself and others have spent the past decade hunting down the data to test these predictions, and so far, he was mostly right, bigger, longer-lived animals do not get more cancer [3–6]. How do these ‘ignorant’ predictions continue to provide insight decades later? Many of the predictions and observations that Peto proposed were rooted in elegant evolutionary theory. Here I propose that cancer research doesn’t need more ignorance, but more theory. Evolutionary biology and ecological theory is a unifying framework that can help us better understand cancer biology [7–10].

## Evolution and ecology can help unify cancer research

The past few decades have led to substantial advances in preventing, diagnosing and treating cancer. Cancer mortality has been declining in the United States, even with our growing population of aging individuals [11]. Cancer prevention and policy has been successful in highlighting important ‘lifestyle’ risk factors, such as, tobacco use, sun exposure, physical inactivity, diet, and oncogenic viruses [12] (i.e., Peto’s black box epidemiology). Screening and access to health care has led to earlier, more precise diagnosis and better outcomes [13], and new treatments have made substantial progress to many cancers [14, 15]. Despite these advancements, cancer is still a major global problem and metastatic disease and therapeutic resistance are considered life-threatening conditions [11]. Peto’s criticism on searching for mechanisms for cancer biology remains salient today, research needs good theory to help guide the right questions. Perspectives from evolution and ecology into cancer research, which have been widely acknowledged [8, 16–19], yet generally under-utilized [20, 21] can provide strong theory and formal models to guide cancer research. Insights from evolution and ecology can explain why we, as humans, mammals and multicellular species, get cancer, as well as provide novel strategies to prevent resistance to therapy, such as adaptive therapy [22, 23].

Cancer is an umbrella term for over three hundred different diseases converging on shared phenotypes, such as sustained proliferation, genomic instability and the evading of the immune system [24]. It’s long been proposed that cancer is a product of somatic evolution [8, 18, 25], where tumor progression parallels diversification and selection in organismal evolution. This stepwise clonal expansion of cells is driven by somatic mutations, which can lead to uncontrolled cellular growth and eventually cancer. However, recent work has demonstrated that phenotypically healthy and non-cancerous tissues can also harbor somatic mutations, including key cancer mutations [26–29]. Further, clonal expansions due to somatic mutations can be found in non-cancerous tissue [30]. Somatic mutations are necessary but not sufficient for cancer development, suggesting we need more than a cellular and/or molecular perspective in cancer research. Open questions remain on why some mutant clones go on to transform into cancer, and why others stay relatively benign clonal expansions. Does this suggest Peto was correct—did the search for the mechanisms in cancer research divert our attention from the causes?

## The curse of ignorance in cancer research

“….genes are like the keys on a piano: Although they are essential, it is the context that makes the music”—Nelson and Bissell 2006 [31]

Somatic mutations and cell proliferation are not the whole story in cancer, nor the sum of its parts. Cancer is part genetic [24], part environment [6, 32], and part bad luck [33]. Although it has been argued luck is more important at the individual level of cancer risk than the population level of cancer risk [34, 35], it’s the interactions between these components that lead to the complex, heterogeneous disease of cancer. Ignoring one component loses insight on the broader complex system, in which the context at the cellular, individual and population level are all contributing factors to disease risk. As such, we need more perspectives (not less) as proposed by Peto, and these perspectives should be rooted in theory to guide our predictions. For example, the distinction between proximate and ultimate causes is an important paradigm in evolutionary biology, and can be a powerful framework to understand disease risk [36]. Proximate causes highlight the immediate mechanisms (i.e., the “how” question) and the ultimate cause emphasizes the adaptive function (i.e., the “why” question). This paradigm was extended and made widely popular by Niko Tinbergen, who added developmental processes and evolutionary history to the existing paradigm [36, 37]. Often called “Tinbergen’s four questions”, this perspective can provide a fuller picture cancer biology and human vulnerability to the disease [38–40] by understanding the proximate causes (i.e., mutations), developmental processes (i.e., accumulation of mutations through aging), evolutionary history (i.e., evolution of multicellular species), and adaptive significance (i.e., how fitness is impacted). These perspectives address different (but not mutually exclusive) needs in cancer research. Understanding the proximate and/or causal molecular mechanisms can hold the key to better diagnosis and innovative treatments, including evolutionary informed treatment strategies [22]. Observations from epidemiology and evolutionary perspectives can lead to successful policy for prevention and early diagnosis. Early diagnosis leads to better outcomes and treatment options. All of these approaches contribute to a transdisciplinary endeavor that is needed in cancer research.

Peto suggested that a strictly molecular-based approach to understanding cancer may be limited in its ability to inform cancer prevention, however, a purely epidemiological approach, by itself, can lead to a similar set of challenges. Peto has previously proposed that “most human cancer is avoidable” [1]. However, targeting epidemiological risk factors only addresses one of the many contributing factors linked to cancer development. The focus on ‘avoidable cancers’ and lifestyle risk factors, such as the link between smoking and lung cancer, is clearly important avenue of research. However, this type of ‘black box epidemiology' framework can lead to misinterpretations of the root causes and could mistakenly focus the blame on the patient. Undeniably, the gap in cancer outcomes is widening based on socioeconomic status [15, 41]. Racial and ethnic minorities have higher incidences of some cancers in the US, which have been linked to inequalities due to structural racism [11]. Individuals have unequal access to clean air, exercise, high-quality diets and healthcare—all of which can contribute to disease risk. While in theory some cancers may be avoidable at a population level, this argument does not apply to individual-level exposure. An individual cannot choose what neighborhood they are born into. Despite the growing interest in research dedicated to understanding the social determinants of cancer risk [42], inequalities in cancer vulnerability persist. There is an increasing need for cancer biologists and epidemiologists to engage with social scientists, policy makers, and individuals that work on social determinants of health to address these gaps in cancer vulnerability across populations.

## Insights from a ‘bigger’ evolutionary framework

As multicellular organisms, we simply can’t avoid cancer. Cancer is evolutionarily ancient and has always been a part of us [43]. Cancer is ubiquitous in multicellular species, including mammals [44]. Many of us may never know we harbor cancerous cells. Malignant cancer has often been found in autopsy reports or scans of individuals that were never diagnosed with cancer [45, 46]. If cancer can be avoidable by studying epidemiological causes, then why do wild animals succumb to the disease as well? Shouldn’t evolution have solved this problem by now?

From the limited data on cancer in wild, free ranging populations of animals, data suggest infectious diseases and environmental toxins can be major contributing factors to cancer in such wild populations [47]. While some argue anthropogenic changes are mostly responsible for cancer in wild populations [48], this cannot explain the presence of tumors in dinosaurs [49, 50], ancient hominin [51], and multiple discoveries of cancer in past human populations [38]. It is unclear how rare cancer was in our past, but there is mounting evidence from paleo-oncology suggesting cancer was ubiquitous [38]. As for the species that are vulnerable to cancer due to anthropogenic change, it’s unclear why certain species are vulnerable to the toxins in their environment, while other species that share the same environment are not [47]. Environmental exposure is not the only contributing risk factor to cancer vulnerabilities. Based on the current data, we can think of environmental exposures as operating a brake pedal, reducing our risk of developing cancer, or stepping on the gas pedal, accelerating the risk of cancer. Even in the lowest risk environments such underlying vulnerability persists.

What mechanism can make our epithelial cells be a million or billion times more cancer-proof than rodents?

Peto’s paradox is not a paradox from a life history perspective. Based on life history theory we expect a Bowhead whale that lives over 200 years to do a better job at maintaining its soma than a field mouse with a lifespan of less than 4 years [52, 53]. Slow-life history animals, such as whales and elephants, have strong selective pressure to maintain their genome—leading to better DNA repair and immune function, which can all contribute to lower cancer risk. Recent work on comparing somatic mutations across mammals provides empirical evidence for this observation. Somatic mutations are evolutionarily constrained and linked with a species lifespan. Longer-lived species, on average, accumulate somatic mutations more slowly than short-lived species. Reducing the mutation rate can substantially decrease risk of cancer [54]. Peto’s paradox, as originally proposed, whereby larger and longer-lived animals do not get more cancer, despite having more cells and a higher probability of mutations, is based on the assumption that somatic mutation rates are equal between mouse and human (leading to equal probability of gaining that cancer causing mutation). However, if somatic mutation rates scale across animals, just a small change (in cancer defense) can have a big effect on protection. In other words, human cells do not need to be a ‘million or billion times more cancer-proof’ if there are fewer mutations to address.

Some studies have begun to shed light on some of the mechanisms responsible for better cancer defenses in long-lived species (reviewed in [55]). For example, long-lived species may have more efficient DNA repair [56], better recognition of DNA breaks [57], more robust telomere protection [58] and heightened adaptive immunity [59]. Additionally, some large, long-lived species have duplicated and expanded tumor suppressor genes [60, 61], such as *TP53* in elephants [3, 62] and there is evidence for positive selection on tumor suppressor and immune function genes [60, 63]. Many of the mechanisms responsible for the lower somatic mutation rates in long-lived animals are still awaiting discovery and have the potential to provide novel insights into cancer treatment strategies.

## Addressing the cat-and-mouse in the room

Is a mouse really more cancer ridden than a human or an elephant? In this reprint, Peto proposed that mouse cells in vitro are very easy to transform into malignant cells and thus this should equate to more cancer in the organism. Indeed, mouse cells accumulate somatic mutations more rapidly [54], supporting Peto’s initial observations. Based on these data, we should conclude that mice get more cancer than humans and elephants. However, recent studies have demonstrated that our small bodied cousins of Rodentia actually get substantially less cancer than carnivores, including the big cats [5, 64]. It should be noted that diet is considered an important risk factor for cancer in human populations, however, broad cross-species observations of cancer are not intended to be interpreted within a species or at the individual level. More empirical work is needed to understand the patterns of diet and cancer risk across species and if this could be translated into similar risk patterns for humans. Overall, current comparative cancer data demonstrates the relationship between body size, lifespan, and cancer risk is not a linear pattern. In other words, somatic mutation rates, species lifespan and environment cannot fully explain cancer risk across the tree of life. This suggests exciting new directions in comparative cancer research, as there may be many solutions of cancer prevention awaiting discovery.

## Cancer is more than a cellular disease

The protective processes probably do lie in the cells rather than in the whole organism…

Mutant subclonal populations are ubiquitous in otherwise ‘healthy tissue’ suggesting cancer is more than just a disease of the genome and cells. Context matters and a multi-level perspective on cancer is necessary. Recent perspectives suggest cancer is a disease of the whole organism [9, 32]. We need to think of cancer in its environment within the host. Cancer is a product of two opposing evolutionary forces—organismal evolution, which favors cellular cooperation of the multicellular body, and somatic evolution, that favors cellular cheaters that outcompete other subclones [65]. The ecology (i.e. microenvironment) of where these rogue cells exist within the organism and the cellular fitness, relative to their neighbor’s fitness, are all important contributors to whether the mutant subclonal lineage will eventually transform into cancer [17, 65]. Future work incorporating ecology and specifically, multiple interacting ecosystems, such as tissue and organismal level tumor control, is a promising next step in cancer research.

## Conclusions

This call for ignorance in cancer research came at a time when molecular biology was dominating the field of cancer. The search for cancer genes, targeted therapies and other mechanism driven research may have muddied the progress towards understanding the bigger evolutionary dynamics of cancer biology. Understanding the mechanisms is an essential step in cancer research, but if every gene can be considered a cancer gene [66], the whole pursuit might seem impractical. However, instead of the proposed ignorance in cancer research, we need more theory [67]. Cancer is a common feature of multicellularity and the important discovery of somatic mutations in key cancer genes in ‘healthy tissue’ is changing how we view cancer. A strictly mechanistic view confines the broader understanding of cancer biology. An evolutionary and ecological perspective unifies these multiple risk factors—allowing for a broader ecosystem—and multi-level- approach to cancer. This multidisciplinary approach needs to incorporate mechanisms, evolutionary and ecological theory, and observations from epidemiology for progress in cancer research. And a good dose of luck…
